# Palliative care nurse specialists’ reflections on a palliative care educational intervention in long-term care: an inductive content analysis

**DOI:** 10.1186/s12904-019-0488-4

**Published:** 2019-11-19

**Authors:** Rosemary Frey, Deborah Balmer, Michal Boyd, Jackie Robinson, Merryn Gott

**Affiliations:** School of Nursing, Faculty of Medical and Health Sciences University of Auckland, 85 Park Road, Grafton, Auckland, New Zealand

**Keywords:** Palliative, Long-term care, Educational intervention, Hospice

## Abstract

**Background:**

Older people in long-term care facilities are at a greater risk of receiving care at the end of life that does not adequately meet their needs, yet staff in long-term care are often unprepared to provide palliative care. The objective of the study was to explore palliative care nurse specialists’ experiences regarding the benefits of and barriers to the implementation of a palliative care educational intervention, Supportive Hospice Aged Residential Exchange (SHARE) in 20 long-term care facilities.

**Methods:**

Reflective logs (465), recorded over the course of the yearlong SHARE intervention by the three palliative care nurse specialists from two local hospices, who were the on-site mentors, were qualitatively analyzed by two researchers utilizing inductive content analysis.

**Results:**

Categories emerging from the logs include the importance of relationships, knowledge exchange, communication, and the challenges of providing palliative care in a long-term care setting.

**Conclusion:**

Evidence from the logs indicated that sustained relationships between the palliative care nurse specialists and staff (registered nurses, healthcare assistants) as well as reciprocal learning were key factors supporting the implementation of this palliative care educational intervention. Challenges remain however in relation to staffing levels, which further emphasizes the importance of palliative care nurse specialist presence as a point of stability.

## Background

As populations age, “home” at the time of death for a growing number of older people will be long-term care facilities [1]. In addition, older people in long-term care are more likely to live with complex co-morbidities and experience illness within the context of existing physical or mental impairment [2, 3]. As one of the most disadvantaged and vulnerable groups in industrial societies, older people in long-term care facilities are thus at a greater risk of receiving care at the end of life that does not adequately meet their needs [4]. The increasingly complex needs of long term care residents and the fact that a large number of older adults will die while long-term care makes it essential that services have processes in place to facilitate quality palliative and end of life care. Long-term care in New Zealand is synonymous with residential aged care. The level of care provided is based on need and includes rest home care (support but not 24 h nursing/medical care), hospital-level care (24-h nursing/medical care), dementia care and psychogeriatric care [5].

The New Zealand Health Needs Assessment for Palliative Care conducted under the auspices of the Palliative Care Council concluded that almost all long-term care facility residents would require palliative care at the end of their life [6]. Furthermore, 50% would benefit from specialist palliative care advice and support, while the other 47% could be managed by the long-term care facility, given the capabilities and resources to provide a generalist level of palliative care [7].

Within the context of this study, long-term care staff refers to registered nurses and health care assistants (non-health professional support workers) directly involved in the care of residents.

Research has indicated that registered nurses in long-term care facilities are often unprepared to provide palliative care [8]. For example, they feel ill-equipped to undertake Advance Care Planning (ACP), [9] a process of discussion and shared planning for future health care that assists the individual to identify their personal beliefs and values and incorporate them into plans for their future health care [10]. There is also evidence that long-term care facility staff (both registered nurses and health care assistants) feel inadequately supported in coping with multiple bereavement experiences [11]. Addressing the palliative care knowledge and skills deficit, as well as the emotional readiness of long-term care facility staff is therefore of critical importance to delivering quality palliative care [7, 12, 13].

However, a major challenge continues to be the translation of educational interventions to the reality of the long-term care environment [14]. The negative impact of burnout on education uptake and the lack of consideration of organisational factors (e.g. low staffing levels, time pressures) may present obstacles to sustainable change [15, 16]. Furthermore, conflicts may arise between hospice as an organization and long-term care facilities hindering the delivery of quality care [17]. The provision of complex, quality health care requires effective relationships among multidisciplinary team members, as well as the ability to learn together and adapt to change [18–21].

Education initiatives developed to date have focused on short training programs concentrating on the traditional “chalk and talk” format [22, 23]. However, there is minimal evidence that nurse and support staff knowledge gained from this format is sustained in the long term [14]. Adults learn best from direct experience [24]. As quoted from Confucius “Tell me, and I will forget. Show me, and I may remember. Involve me, and I will understand.” It is within this context that the need for a new model of education delivery has been identified that supports “hands-on” learning which is a vital component of the sustained transfer of new knowledge into practice [25].

The Supportive Hospice Aged Residential Exchange (SHARE) intervention provides a means to package and systematically support knowledge exchange between hospice palliative care nurse specialists and long-term care facility direct care staff (registered nurses, health care assistants). Hospice palliative care nurse specialists are defined as registered nurses with a recognized palliative care qualification [26] Although hospice involves in-patient services, hospice palliative care nurse specialists also provide care in the community including to residential aged care residents [27]. The goal of SHARE is to improve palliative care delivery [28]. Palliative care in this paper is defined as an approach to care that improves the quality of life of patients and their families for those facing a life-threatening illness [29]. It involves care across the duration of the resident’s illness [29]. End-of-life care is incorporated into palliative care, although the timeframe differs in that it is typically limited to the last few months of life [30]. SHARE implementation involved weekly visits by one of three palliative care nurse specialists from two local hospices to twenty local long-term care facilities.

*SHARE components.* The SHARE model included: 1) a records review and assessment of the goals of care of residents identified as having palliative care needs by the palliative care nurse specialist and facility RN, using the Clinical Frailty scale [31] and Supportive Palliative Care Indicators Tool [32]; 2) palliative care nurse specialist and RN reciprocal clinical coaching; 3) role modelling of advance care planning conversations with RN’s; 4) palliative care education planning and 5) debriefing following a resident death with the facility RN’s, and to a lesser degree with HCA’s [28]. Evidence indicates that these methods can achieve sustained knowledge transfer [32–34]. (Table 1). SHARE was implemented and evaluated in 20 long-term care facilities, for 1 year in two district health boards. Each palliative care nurse specialist was assigned a subset of the 20 facilities to visit weekly.

**Table 1:** Components of the SHARE model (Frey et al., 2017)

Records Review	
The identification of residents who would benefit from a palliative approach was completed through a records review conducted by the hospice palliative care nurse specialist in conjunction with a registered nurse (RN) from each facility. The review included an assessment of resident palliative care need using the Supportive Palliative Care Indicators Tool [31] and the Clinical Frailty Scale [32]. The purpose of the review was to provide the basis for ongoing monitoring of resident palliative care need and to create a “Goals of Care” plan for those on the registry.	
Clinical Coaching and Role Modelling	
This was a reciprocal process of shared learning between palliative care nurse specialists and long-term care RN’s and healthcare assistants (HCA’s). In partnership with HCA’s, RN’s and General Practitioners (GP) the palliative care nurse specialists worked to develop and update a “Goals of Care” plan to reflect new or changing palliative care needs. This consultation was made in partnership with the RN and HCA present to provide opportunities for clinical coaching, role modelling and development of clinical knowledge.	
Palliative Care Education Planning	
The palliative care nurse specialist worked together with RN’s and HCA’s to discuss the specific learning needs in each facility identifying the priorities for staff. A programme of education was be developed that was unique to that facility and complimented the current education provided by the two hospices.	
Debriefing	
Debriefing following resident deaths was offered facilitated by the palliative care nurse specialist in collaboration with a senior RN from the facility. This service provided an opportunity to acknowledge the emotional impact of end of life care. It also provided an opportunity to reflect on the care provided.	

The goal of the larger SHARE evaluation was to determine if the intervention was contextually appropriate and sustainable. This study forms *Phase One* of the larger SHARE evaluation and explores the palliative care nurse specialists’ views and experiences regarding the benefits and barriers to SHARE implementation in long-term care facilities. The effective implementation of any educational intervention is dependent on the perceptions and interpretations that both mentors and students bring to the encounter [33]. While there are studies focusing on the impact of palliative care interventions on residents, clinical staff and families [34–37] little research has dealt with the perceptions of the facilitators [38]. An individual’s perceptions of experience directly influence their subsequent motivation [39]. It would seem important that the perceptions of the palliative care nurse specialists delivering SHARE be examined.

## Methods

The objective of the study was to explore hospice nurses’ views and experiences regarding the barriers and facilitators to the implementation of a palliative care educational intervention (SHARE) in long-term care facilities. The study forms part of a larger yearlong mixed-methods evaluation of SHARE in 20 long-term care facilities.

### Participants

All of the palliative care nurse specialists were female and between 40 and 55 years of age. One palliative care nurse specialist had between five and 9 years of experience in the field while the other two had over 10 years’ experience in palliative care. Two of the palliative care nurse specialists had less than 5 years’ experience working with long-term care while one had over 20 years working with the sector. Given the small number of contributors, identifiers for quotes (log ID and facility identification) have been omitted to maintain the confidentiality of both the palliative care nurse specialists and the facilities.

### Process

The extensive reflective logs, recorded over the course of the yearlong SHARE intervention by the three palliative care nurse specialists, who were the on-site mentors, were qualitatively analyzed. The palliative care nurse specialists were asked to keep a weekly journal for each of the 20 facilities. The journals included sections on resident care as well as a section for reflective comments about their perceptions of how the intervention protocol supported the goal of improving palliative care knowledge among staff in long-term care facilities. Journals were selected as the most appropriate method for capturing a personal in-depth perspective on a day to day basis [40]. Their contemporaneous nature makes journals less subject to the effects of context and recall biases than other data collection methods such as interviews [41]. Ross et al. [42] in exploring the use of diaries (journals) by nurses concluded that this tool was a “trustworthy and effective method of data collection (p. 417)’ This report focusses on the reflective section. Four hundred sixty-five reports (average of 23/facility) were submitted by the palliative care nurse specialists over the 1 year of SHARE implementation. All data collection took place between September 2017 and November 2018. The start of SHARE in facilities was staggered across the period. Significant information obtained from the journals included palliative care nurse perceptions of improved registered nurse communication with families, improved registered nurse identification of residents with palliative care needs, palliative care nurse specialist gains in knowledge in gerontology and dementia care.

### Analysis

All logs were imported into QSR NVivo 12 for analysis. The process of inductive content analysis was utilized [43]. The researchers (RF, DB) read all logs several times. Open coding headings were used to describe all aspects of the content. Headings were listed on a coding sheet and categories were created. Following open coding, the categories were grouped together into higher-order headings by combining categories with similar content. Higher-order headings were refined as the analysis process progressed through in-depth discussions between DB and RF. The names of the categories were selected based on their ability to represent the overall sense of the logs. Subcategories were identified through the process of abstraction.

## Results

**Fig. 1:**
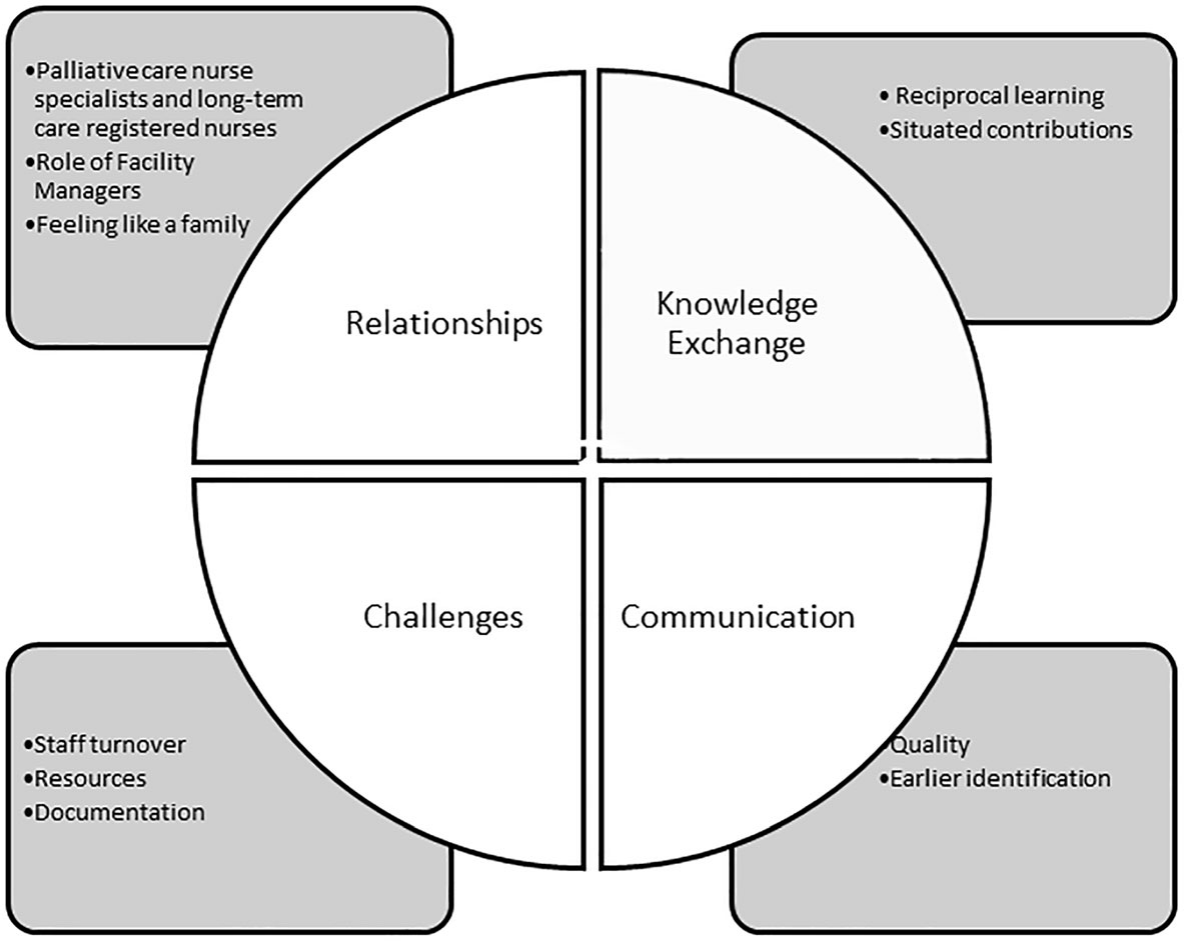
Categories and sub-categories identified within the log texts

Four categories were isolated. Three categories reflected the benefits of SHARE (relationships), knowledge exchange, communication), while barriers to SHARE are reflected under the category challenges. Categories and subcategories are portrayed in Fig. 1.

### Relationships

A common topic from all the logs was the building of relationships – of facility staff with residents, and with families, connections among facility staff, and in the case of the hospice nurses, the importance of building a strong connection with the facility staff in order to build trust.

#### Palliative care nurse specialists and long-term care registered nurses

The establishment of relationships between the palliative care nurse specialists and long-term care facility registered nurses, helped develop a sense of both trust and understanding of the role and scope of hospice in the care of residents.

*SHARE helped the long-term care facility team and myself developing a better rapport. Particularly in my relationship with care staff, they would share with me about their wedding anniversaries and family’s struggles. I sensed that there was added meaning to their work somehow.*

The following quote highlights the need for the reinforcement and support provided by the palliative care nurse specialist as illustrated by this example of the isolation within which facility registered nurses often work.

*Long-term care facility nurse was working in such a lonely environment. I could see the joy in the eyes of RN’s [registered nurses] [name] and [name] when I listened to them. When I gave RN [name] my honest praise about her kindness and compassionate care to her elderly resident, she actually had tears. She was very humbled and said, “I was doing my job. I thought I was doing what everyone does!” Such beautiful and caring nurses!*

#### Role of facility managers

Support for SHARE on the part of facility managers fostered closer relationships between registered nurses, health care assistants, and managers. The strengthened relationship managers, in turn, facilitated greater collaboration with the palliative care nurse specialists:

*One [health care assistant] in particular whom I found to be very prickly when I first started visiting [facility] and how her demeanor had changed over the last 12 months. When I walked in today, I could hear her laughing and joking with the staff and she even came up and gave me a hug. I put that down to the way that [manager] has been managing and supporting the staff and the improvements she has made within the facility.*

#### Feeling like a family

Opportunities for participation in activities encouraged socialization between facility staff members (RN’s, HCA’s) and between staff and residents. These activities provided a mutual feeling of “family” that fostered the development of collaborative relationships, a key prerequisite for SHARE success.

*It was clear which facility did want to involve residents ‘family.’ When Nurse Manager A announced Christmas decoration competition in individual Suites, I could hear that the focus was on the dignity of residents … Looking at the staff involvement in their own party and the trolley filled with Secret Santa, I felt this place was full of love…If staff were not happy, they wouldn’t bother to participate in Secret Santa or Share lunch.*

### Knowledge exchange

#### Reciprocal learning

The sharing of palliative care knowledge and applied between the hospice nurses and the long-term care facility staff and growth in hospice nurses’ understanding of geriatric care was evident in the logs. The hospice nurses’ perspectives on care in long-term care facilities revealed new understandings of geriatric care and the palliative care trajectory of chronic illness with all of its uncertainty.

*The learning from SHARE discussions with [name] [RN] has been most beneficial to inform my knowledge to a broader level of the “strength of the human spirit to survive when the body and mind are dying” especially with reference to those who have dementia and advanced frailty.*

#### Situated contributions

There is considerable evidence of situated interventions by the hospice nurse where the hospice nurses’ deliberate presence in the long-term care facility created plentiful moments where discussion, learning, teaching, and changes in practice took place.

*It was quite rewarding to see RN [name] come up with the resident for Palliative Care Register. This RN team worked quietly and never showed any excitement on the SHARE visit. Thus, when they voluntarily gave me a name “[Hospice Nurse], I think this resident was ready for a palliative care approach …*” *I was quietly joyful. Without SHARE, this facility had not been offered palliative care education in the past and (a hospice) community team had not been in touch for at least 2 years.*

Other insights for the hospice nurse indicated areas for further education. This was particularly true in regard to maintaining ongoing communication with families about changes in their relative’s condition and care needs.

*I was surprised that the predominance of residents reviewed was by default as*” *For Resus” [resuscitation] … RN/facility under the impression of the resident is unable to cognitively decide rendered them as ‘resuscitatable’ rather than working with the family as part of ACP [advance care planning] process.*

### Communication

#### Quality

Registered nurse confidence in communication skills with regard to palliative and end of life care as well as the ability to initiate advance care planning with families improved in some facilities. One hospice nurse reflected on both the improved palliative care knowledge and communication skills of a registered nurse:

*The long-term care facility team had been very caring for him [resident] and kept close communication with him [resident] and his son. RN [name] was able to share with me that the resident [name] and his son discussed funeral arrangements in the last 48 h. She felt particularly proud of able to recognise dying and facilitate communication amongst resident and his son.*

#### Early palliative care needs identification

The logs also provided evidence of better documentation of residents with palliative care needs. Better identification allows for proactive care planning before the terminal stage.

*When I was preparing for the resident’s information for a research report, I realised that all recent deceased residents were enrolled onto Palliative Care Register.*

### Challenges

Key themes reflected throughout the logs included the detrimental effects of resource constraints and increasing staff turnovers. These factors not only influence palliative care education and delivery but also staff well-being.

#### Staff turnover, & under-resourcing

The level of reference to staff turnover, insufficient staffing & staff changes (especially RNs coming in from overseas who use the long-term care sector to bridge into work in district health boards) is troubling. This, in turn, led to very challenging circumstances in which to provide staff education in a traditional classroom sense, making the physical presence of the hospice nurses even more significant in sharing knowledge and practice between the long-term care facilities and the hospice nurses.

*The lead clinical nurse in the Dementia Unit has left. Find this unfortunate as she appeared to us to be very knowledgeable in the care of those with dementia. They do not have someone to replace her as yet.*

The continuing staff shortages serve as a further indicator of the need for an alternative to traditional didactic methods of teaching. Staff shortages translate into a lack of staff available to attend sessions, as indicated in the following reflection:

*Even CCM [clinical charge nurse manager] A could honestly share that she was constantly orientating a new group of nurses. They were not in any shape to take on [education] training at all.*

#### Documentation

Problems persisted in some facilities particularly in relation to future care documentation. The palliative care nurse specialists in the SHARE study documented practices that they found troubling concerning palliative care and especially unnecessary hospital admission.

*A new nurse was on duty and there were no clear easy to access guidelines in residents’ notes about her future plan of care. Therefore, by default, she went to the hospital where she spent 24 h, was commenced on oral antibiotics and then returned to the facility.*

## Discussion

A number of factors supported the educational intervention, Supportive Hospice Aged Residential Exchange (SHARE) as perceived by the three hospice nurses. In the first instance, the relationship that the palliative care nurse specialists forged with facility registered nurses, health care assistants as well as facility managers appeared to have a huge bearing on the success of the uptake of the learning. Developing a connection and acceptance of the specialist nurse specialist was key – i.e. that the palliative care nurse specialist needed this relationship to be developed in order to feel her role was effective. Indeed, previous research has indicated a relationship between improved student outcomes and the development of a trusting teacher-student relationship [44]. Having a dedicated palliative care nurse specialist visiting on-site regularly allowed the registered nurses to build a key relationship, encouraging them to share the gaps in their knowledge, as well as to ask for support in working with families. Comments on the personal support that the palliative care nurse specialist gave indicate that along with providing specialist palliative care knowledge, they became a source of comfort for many stressed registered nurses. Trust has also been associated with increased sharing and collaboration [45]. In fact, trust and collaboration reinforce each other [45]. Ongoing contact between the parties (in this instance, palliative care nurse specialists, registered nurses and health care assistants), creates the opportunity to increased trust, leading to enhanced motivation to learn [46]. This increased motivation, in turn, supports a willingness for continued collaboration. In other words, with ongoing contact, the palliative care nurse specialist gained acceptance within the facilities and was in turn welcomed as part of the “staff family”. The development of a trusting relationship where registered nurses felt “safe” to ask for help with caring for residents with palliative care needs was a key component of the SHARE model. Relationships between staff (RN’s, HCA’s) and facility managers were also key to palliative care nurse specialists’ perceptions of improved resident and family care. Previous research has indicated that the quality of the relationships and communication among staff members is a key predictor of health care quality [47].

Drawing on Lave and Wenger [48], learning within long-term care facilities is a situated and collaborative activity, a process of participation in “communities of practice.” Learning is context-bound, shaped by the sociocultural practices of the organization. Indeed, research indicates that setting, activities, and artifacts also play a key role in learning, particularly in tasks that require higher-order knowledge [49]. According to Billet [49], “the adaptability of the knowledge that has been learned is premised upon its discernible applicability to particular situations” (p. 389). Findings also point to evidence of reciprocal learning with palliative care nurse specialists gaining new knowledge and understanding during the interactions with registered nurses and health care assistants.

Previous research has indicated that mentoring is linked to personal and professional development for mentors [50]. Palliative care nurse specialist mentors appeared to have expanded their knowledge of gerontology as well as their understanding of long-term care registered nurse perspectives [51]. In essence, the palliative care nurse specialists and the registered nurses in the long-term care facilities developed a peer-learning partnership – a reciprocal learning relationship between parties of equal status who share a common goal [52]. Findings indicated that interactions as part of the SHARE role increased the palliative care nurse specialists’ respect for the care provided by the facility as well as their own knowledge and skill to care for frail older people. The partnership thus facilitated knowledge exchange between the palliative care nurse specialists and registered nurses and health care assistants with the goal of improving palliative care delivery within the long-term care facilities. This, in turn, helped to establish a trusting relationship built on mutual respect [53].

Evidence from the hospice logs indicated both a recognition of improved communication about changes in a resident’s condition with family members. Excellent palliative care occurs when interdisciplinary team members communicate effectively and collaborate on care plans [34]. Therefore, it is necessary for all health care providers (including health care assistants) to become more effective at interpersonal communication and collaborative skills [54]. Mentoring by palliative care nurse specialists appears to have enhanced interpersonal communication skills for registered nurses. Barriers to communication persisted, however, particularly in relation to the initiation and documentation of advance care plans for residents. There is a significant relationship between advance care plans and quality of dying [55] as well as a relationship between the care received at end of life and patient preferences [56]. As in previous research, lack of willingness to document palliative care need may stem from prior uneasiness with discussing advance care plan related issues with residents or families [57].

The level of reference to staff turnover, insufficient staffing, and staff changes was highlighted by hospice nurses and represented a barrier to SHARE implementation. Low staffing levels and the associated time pressures can create barriers to the uptake and application of new knowledge [58]. Previous research has indicated a staff preference for interactive, hands-on, applied learning [59] making the physical presence of the palliative care nurse specialists even more significant in sharing knowledge and practice. Furthermore, traditional training and education methods in palliative and end of life care have previously required registered nurses to leave the clinical environment to attend study days and training sessions [60] creating more staffing pressures for long-term care facilities. In contrast, SHARE does not pull registered nurses away from the bedside and therefore does not require “more time” to attend teaching sessions. Nevertheless, the continued staff turnovers presented a challenge to the establishment of trusting relationships between the palliative care nurse specialists and new staff registered nurses. Such challenges require the development of skills on the part of palliative care nurse specialists to create genuine connections, even in brief encounters. Such skills can result in greater trust and opportunities for teamwork to develop [61].

### Strengths and limitations

The findings and consequent discussion are based solely on the perceptions and observations of the hospice nurses. The views and opinions of others involved in the evaluation, such as the long-term care facility registered nurses, managers, and residents, have not been included. However, because the logs were maintained over the course of a year, emerging patterns were revealed which may not have been observable with other methods. Furthermore, both contextual and recall biases were significantly reduced as logs were created as events unfolded [62].

## Conclusion

The overall impression of the palliative care nurse specialists was that SHARE supports the building of a strong relationship between the hospice nurse specialists and facilities, facilitates improved communication between registered nurses and residents and registered nurses and families and alerts registered nurses to be vigilant in assessing the palliative care needs of their residents. Furthermore, evidence from the logs indicated that the more the palliative care nurse specialists interacted with long-term care registered nurses and health care assistants, the better the knowledge base for both sides. Role modeling of difficult conversations with families may build confidence in the long-term care registered nurses to begin advance care planning conversations earlier as well as improve ACP documentation. Barriers to SHARE implementation remain however in relation to long-term care staffing levels and staff turnover. Ultimately, continued implementation of SHARE and the form of that implementation are dependent on both resourcing and the commitment of all parties involved. However, the hospices, district health boards and long-term care decision-makers are committed to ensuring effective and timely knowledge transfer from evidence gathered in this study into policy and practice within long-term care.

## Data Availability

The dataset used and/or analysed during the current study are available from the corresponding author on reasonable request in a de-identified form.

## Abbreviations

ACP: Advance care planning
CCM: Clinical charge nurse manager
HCA: Healthcare assistant
RN: Registered nurse
SHARE: Supportive Hospice Aged Residential Exchange
